# Developing a framework for promoting interest and engagement of scholarship of teaching and learning for medical students

**DOI:** 10.1080/10872981.2024.2336332

**Published:** 2024-04-01

**Authors:** Ritvik Bhattacharjee, Austin Reynolds, Lilian Zhan, Laura Knittig, Ranjini Nagaraj, Yuan Zhao

**Affiliations:** College of Osteopathic Medicine, Sam Houston State University, Conroe, USA

**Keywords:** Scholarship of teaching and learning, medical education research, medical student research, student-faculty partnership, academic medicine

## Abstract

**Background:**

The scholarship of teaching and learning (SoTL) is a field of academic research that focuses on improving learning through reflective and informed teaching. Currently, most SoTL-related work is faculty-driven; however, student involvement in SoTL has been shown to benefit both learners and educators. Our study aims to develop a framework for increasing medical students’ interest, confidence, and engagement in SoTL.

**Methods:**

A student-led SoTL interest group was developed and a year-round program of SoTL was designed and delivered by student leaders of the group under the guidance of a faculty advisor. Individual post-session surveys were administered to evaluate participants’ perceptions of each session. Pre- and post-program surveys were administered to evaluate the program impact.

**Results:**

The year-round SoTL program consistently attracted the participation of medical students and faculty. Survey responses indicated strong medical student interest in the program and positive impact of the program. Increased interest and confidence in medical education research were reported by the student participants. The program design provided opportunities for student participants to network and receive ongoing feedback about medical education research they were interested or involved in.

**Conclusion:**

Our study provides insights for developing a framework that other institutions can reference and build upon to educate and engage students in SoTL.

## Introduction

SoTL is a specialized research field that systematically investigates the effects of pedagogy on student learning outcomes [1]. Health professions educators have embraced the value of SoTL [2–4]; however, SoTL-related scholarly projects are predominantly faculty-led, though students are critical stakeholders in the education process and play a key role in the evaluation necessary for quality improvement [5–7]. In recent years, there has been a call to expand students’ role in SoTL to advance the growing field [7–9]. Despite increased recognition of the value that students bring to SoTL, literature is scarce for structured frameworks that support the growth of student SoTL researchers, particularly in medical schools. Thus, we developed a framework for a SoTL interest group and program for medical students and evaluated its impact on their interest and confidence pertaining to SoTL research.

## Methods

A student-led SoTL interest group was developed in summer of 2021 by two rising second-year medical students, who were interviewed and selected by the faculty advisor per their interest and experience. A year-round program containing workshops and events was designed by these students with guidance from the faculty advisor. The curriculum for the workshops was developed with guidance from a SoTL textbook [10].

The program began in fall of 2021 and was updated in 2022 based on feedback from the previous year, which mainly included better scheduling and the addition of a student research presentation session. In 2022, new student leaders (*n* = 3) were selected from active participants in the 2021 cycle by the faculty advisor and delivered the program similarly to the inaugural leaders. A total of five workshops, two events, and an abstract competition were delivered to the SoTL interest group to meet the curriculum objectives (Table 1). Each program session lasted one hour and was delivered with active learning methods including small- and large-group discussion, scenario-based learning, and worksheet activities. First- and second-year medical students (*n* = 252) and faculty (*n* = 32) were invited.

**Table 1.** Curriculum sequence, session plan, and schedule of the year-round SoTL program.

Date	Topic of Workshop or Event	Session Plan
Fall Semester		
September	Introduction to SoTL	Participants gain an overview of the process outlining SoTL research and the program goal and plan. Faculty with SoTL interest share experience and engage with students in round table discussion to brainstorm general SoTL research themes.
October	Medical Education Research Presentations	Previous students present their SoTL work and engage conversation with participants regarding the development of SoTL research projects.
November	The Art of the Research Question	Student and faculty participants work through a worksheet to identify research theme and a specific research question. Key works are also identified for literature review.
December	Research Methodology	Participants learn the quantitative and qualitative methods for conducting SoTL research and work to apply knowledge to help design their studies.
Spring Semester		
January	Faculty-Student Research Luncheon	Round table luncheon is organized to connect students and faculty of the same research interest (other research types were also invited)
February	Creating Your Grant Proposal	Participants learn about the components and writing strategy of research proposal as well as funding resources pertaining to SoTL research. Funded proposals are provided to individual participant to engage discussion.
April	How to Write Abstracts and Create Posters	Participants work through abstract and poster examples to gain knowledge for abstract writing and poster presentation.
May	Abstract Competition	Student participants have opportunity to submit an abstract of SoTL project they work on per their learning in the program. Faculty advisor selected the winner based on quality of the work and monetary award was presented to the winners.

Student participants were surveyed before they attended their first session in the program and at the end of the program. (Appendix 1) Student participants were also surveyed at the end of each workshop or event to evaluate their perception, familiarity, and interest in SoTL and the program. Participation in the program and surveys was completely voluntary. Descriptive statistics were used to analyze the data and pre- and post-program survey results were compared using a one-tailed t-test in Excel.

Exempt status for the research project was granted by the IRB committee of Sam Houston State University.

## Results

The year-round SoTL program consistently drew the engagement of both medical students and faculty (Table 2). A total of 55 medical students attended at least one session in the program throughout the 2022–2023 school year. Among them, six attended at least half of the sessions. A post-event survey was administered to student participants at the end of each workshop or event (except for the research luncheon and abstract competition). The majority of survey participants responded with ‘agree’ or ‘strongly agree’ to the three statements related to increased familiarity and interest of SoTL and the program (Table 3).

**Table 2.** Attendance of the year-round SoTL program.

Event	OMS1 (N)	OMS2 (N)	Female Students (N)	Male Students (N)	Total Students (N)	Total Faculty (N)
Introduction to SoTL	7	4	5	6	11	9
Medical Education Research Presentations	14	10	14	10	24	10
The Art of the Research Question	9	2	1	10	11	9
Research Methodology	3	9	3	9	12	4
Creating Your Grant Proposal*	8	2	6	4	10	1
How to Write Abstracts and Create Posters*	9	0	5	4	9	1

OMS1: Osteopathic Medical Student-Year One.

OMS2: Osteopathic Medical Student-Year Two.

*Faculty were not invited to the workshop due to class schedule. Only faculty advisor attended the session.

**Table 3.** Perception of individual event of the SoTL program.

Event (Number of Survey Participants)	I am more familiar with SoTL. (Mean/SD)	I am more interested in SoTL. (Mean/SD)	I would attend an event like this in the future. (Mean/SD)
Introduction to SoTL (6)	4.67 (0.47)	4.83 (0.37)	5 (0)
The Art of the Research Question (6)	4 (1.41)	4 (1.41)	4 (1.41)
Research Methodology (8)	4.5 (0.5)	4.5 (0.71)	4.5 (0.71)
Medical Education Research Presentation (19)	4.37 (0.48)	4.47 (0.5)	4.47 (0.5)
How to Write a Grant Proposal (8)	4.38 (0.48)	4.25 (0.43)	4.63 (0.48)
How to Write an Abstract and Create a Poster (5)	4.8 (0.4)	4.6 (0.8)	4.8 (0.4)

The pre- and post-program survey responses indicated significantly increased interest of student participants in getting involved in medical education research and in their confidence in developing educational research questions, evaluating medical education literature, and overall abilities as researcher (Table 4). Student participants also reported significantly increased opportunities for research networking and receiving ongoing feedback through the program.

**Table 4.** Perception of individual event of the SoTL program.

	Pre (*n*=12) Mean (Std)	Post (*n*=13) Mean (Std)	P value (one-tailed)
I am interested in carrying out educational research in my future career.	3.42 (0.9)	3.92 (1.14)	0.12
I am likely to become involved in a medical education research project in the upcoming year.	3.75 (1.06)	4.46 (0.75)	0.035*
I feel confident in my ability to develop research questions for medical education projects.	2.75 (0.97)	4.15 (10.94)	0.0008*
I feel confident in my ability to critically evaluate education research literature.	3.25 (1.06)	4.31 (0.91)	0.008*
I have been able to network with peers with similar research interests in medical education.	2.92 (1)	4.23 (0.9)	0.001*
I have had opportunities to receive on-going feedback about medical education research I’m interested or involved in.	2.58 (0.9)	4.15 (0.9)	0.0002*
I feel confident in my overall abilities as a researcher.	3 (1.04)	3.92 (1.27)	0.03*

## Implications

In our project, we established an innovative framework in which a SoTL program was developed and delivered through a student-led SoTL interest group. Previous study on SoTL programs specifically for students was limited to graduate students only [11]. To our knowledge, our program has a unique design to include medical students directly in the planning and execution of a SoTL program specifically for medical students. This design not only integrates peer teaching concepts to better engage student participants in SoTL learning, but also provides the opportunity to develop student leaders’ essential skills for an academic career. Our data shows very positive perceptions of the program from the student participants and suggests that such framework can increase medical students’ interest and confidence in carrying out SoTL research. Although we did not evaluate faculty participants’ perception, their engagement in the program was critical for making connections with and providing feedback to the student participants.

We found several keys to the success of the program. First, active learning through interactive workshops rather than more traditional, ‘lecture-style’ presentations resulted in better student engagement. Second, strategically scheduling the workshop and events to work around exam schedules helped improve attendance at events. Third, administrative support, such as providing funding for the program, is helpful although not absolutely required. In our project, we have been able to purchase student membership for IAMSE, SoTL textbooks, gift cards for abstract competition winners, and food for the events. Finally, the student leaders of the SoTL interest group play a critical role and need to be carefully selected for strong SoTL interest, communication, and organizational skills.

Our study is limited by the lack of longitudinal tracking of student participants’ research productivity derived from the participation of the program. This will be included in a future study to evaluate the overall success of the program in enhancing medical students’ involvement in SoTL research and expanding their roles as collaborators and partners. The program can also be improved by integrating project-based learning to help students carry out a SoTL project directly in the program. We believe that institutions that have pride in their mission of teaching will place value in SoTL to maintain excellence in the education they offer to their students. Our study has suggested that the framework we developed is practical and duplicable for other medical schools to build on and tailor to their students per the unique culture and mission.

## Data Availability

The datasets used and/or analyzed during the current study are available from the corresponding author on reasonable request.

## Appendix 1. Pre- and Post-Program Survey

1. What is your graduating class?

A. Class of 2024 (OMS-III)

B. Class of 2025 (OMS-II)

C. Class of 2026 (OMS-I)

### C.

2. What is your gender?

A. Female

B. Male

C. Non-binary

D. Prefer not to answer

3. What is your age group?

A. 18-25 years

B. 26-30 years

C. 31-35 years

D. > 35 years

4. What is your incoming undergraduate major?

A. Science, Technology, Engineering, and Math

B. Liberal Arts, Fine Arts, and Business

C. Combination of those listed above

D. Other

5. How many research hours did you list on the TMDSAS application?

A. None

B. <200 hours

C. 200-500 hours

D. >500 hours

6. Please select any of the following that you have prior research experience in:

- Evaluating literature
- Designing research methodology
- Conducting experiments
- Writing an abstract
- Creating a poster presentation
- Participating in a journal club
- Writing a manuscript
- Contributing to a grant proposal application
- Contributing to an IRB application
- Other
- Not applicable

7. Do you have an idea for a novel scholarly project involving medical education outcomes?

A. Yes

B. No

8. Are you currently working or have worked in the past on a scholarly project involving medical education outcomes?

A. Yes

B. No

9. Please read through the following statements and rate your agreement on a scale from 1–5. (1-Strongly Disagree, 2-Disagree, 3-Neutral, 4-Agree, 5-Strongly Agree)

- I enjoy research.
- I plan to pursue a career in an academic setting.
- I am interested in carrying out educational research in my future career.
- I am likely to become involved in a medical education research project in the upcoming year.
- I feel confident in my overall abilities as a researcher.
- I feel confident in my ability to develop research questions for medical education projects.
- I feel confident in my ability to critically evaluate education research literature.
- I have been able to network with peers with similar research interests in medical education.
- I have had opportunities to receive on-going feedback about medical education research I’m interested or involved in.
